# Noninvasive Recanalization of a Coronary Chronic Total Occlusion

**DOI:** 10.1155/2019/7979316

**Published:** 2019-04-10

**Authors:** Nikola Kos, Vjekoslav Radeljić, Nikola Pavlović, Krešimir Kordić, Kristijan Đula, Nikola Bulj, Tomislav Krčmar, Diana Delić Brkljačić, Ivan Zeljković, Šime Manola

**Affiliations:** Department of Cardiovascular Diseases, University Hospital Centre Sestre Milosrdnice, Zagreb, Croatia

## Abstract

**Background:**

Spontaneous recanalization of a chronically occluded artery is rare and reported anecdotally.

**Case Summary:**

We report a case of a patient with a chronically occluded right coronary artery, found on a coronary angiography performed due to acute ST elevation myocardial infarction with an occluded circumflex artery as a culprit lesion. Three months later, a follow-up angiography was performed and a recanalization of the occluded right coronary artery was detected.

**Discussion:**

There is a possibility that intrinsic fibrinolytic mechanisms with the additional effect of standard antithrombotic drugs administrated after the acute coronary event led to the recanalization.

## 1. Introduction

Chronic total occlusion (CTO) of a coronary artery is defined as an occlusion > 6 weeks with well-developed collaterals which may provide flow equivalent to a flow obtained in an artery with 90-95% stenosis, preventing myocardial ischemia [1–4]. Although a spontaneous recanalization can occur in the acute myocardial infarction caused by a thrombotic event, it is a rare event in CTOs, especially in native coronary arteries occluded by a solid fibrocalcific plaque. We report a case of a female patient with a spontaneously recanalized CTO of the right coronary artery (RCA).

## 2. Case Report

**Figure 1:**
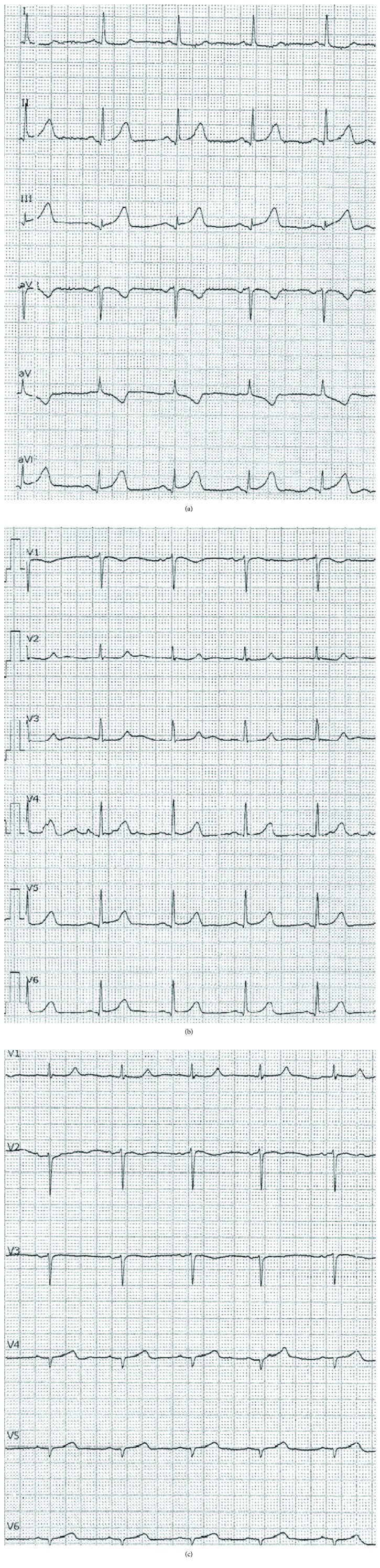
(a) ECG at admission (I, II, III, aVF, aVL, and aVF). (b) ECG at admission (V1-V6). (c) ECG at admission (right leads).

**Figure 2:**
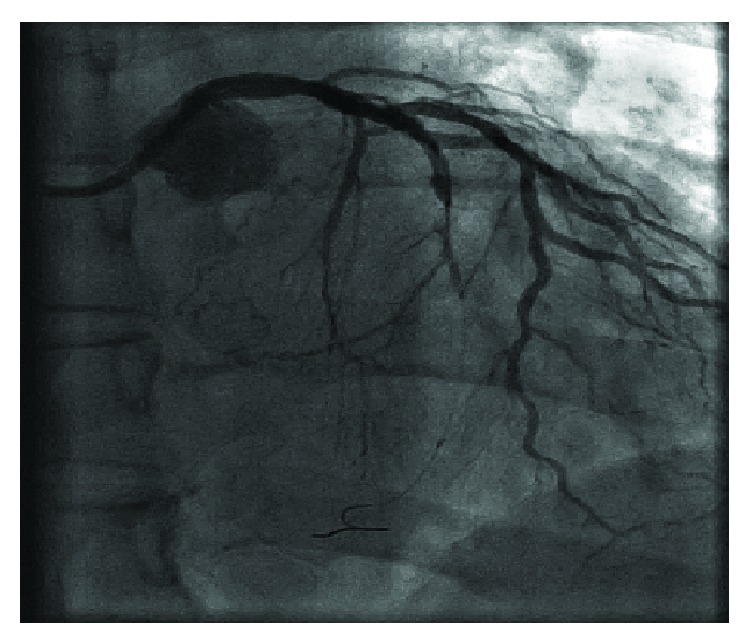
Occlusion of the middle segment of the left circumflex artery (LCx) (image after a partial recanalization with a guidewire).

**Figure 3:**
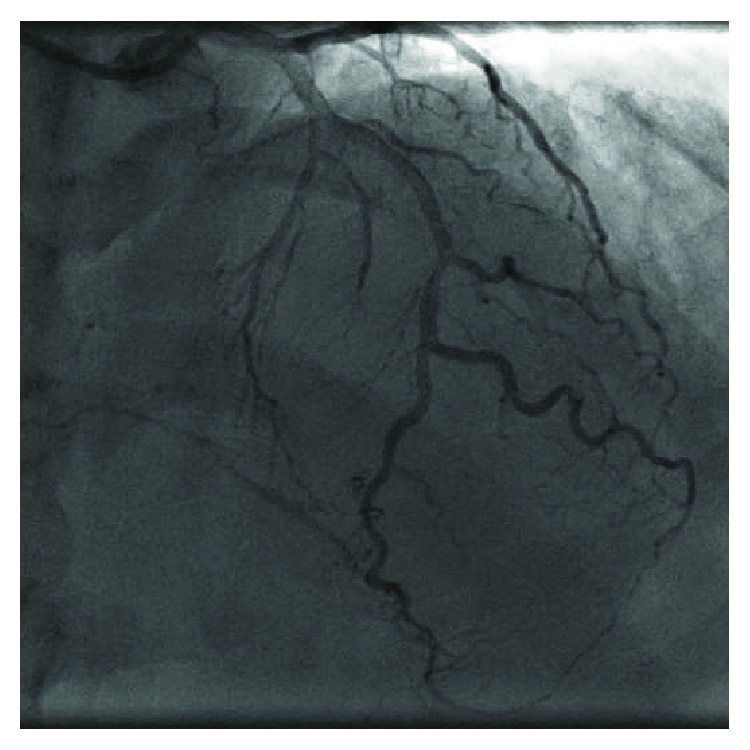
Left anterior descending artery (LAD) with collaterals to the right coronary artery (RCA).

**Figure 4:**
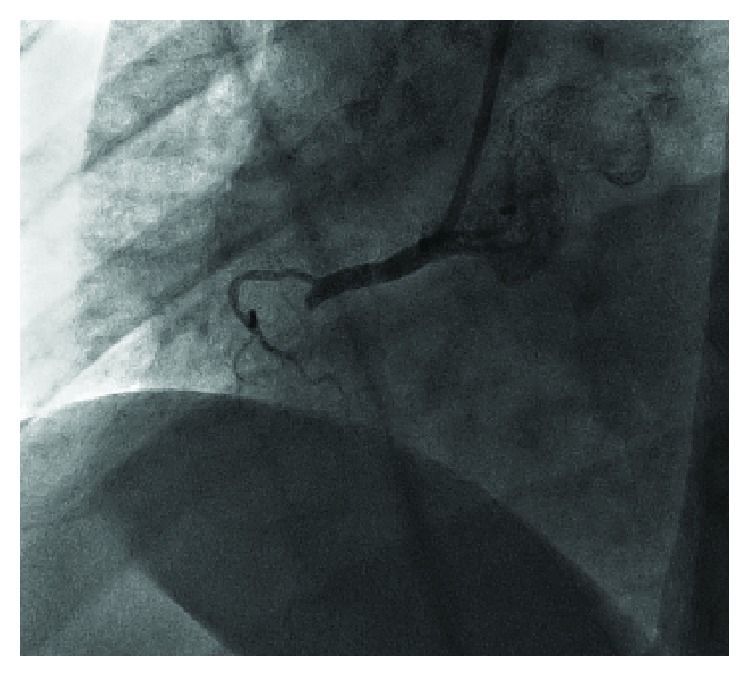
Chronic total occlusion (CTO) of the proximal right coronary artery (RCA) segment.

**Figure 5:**
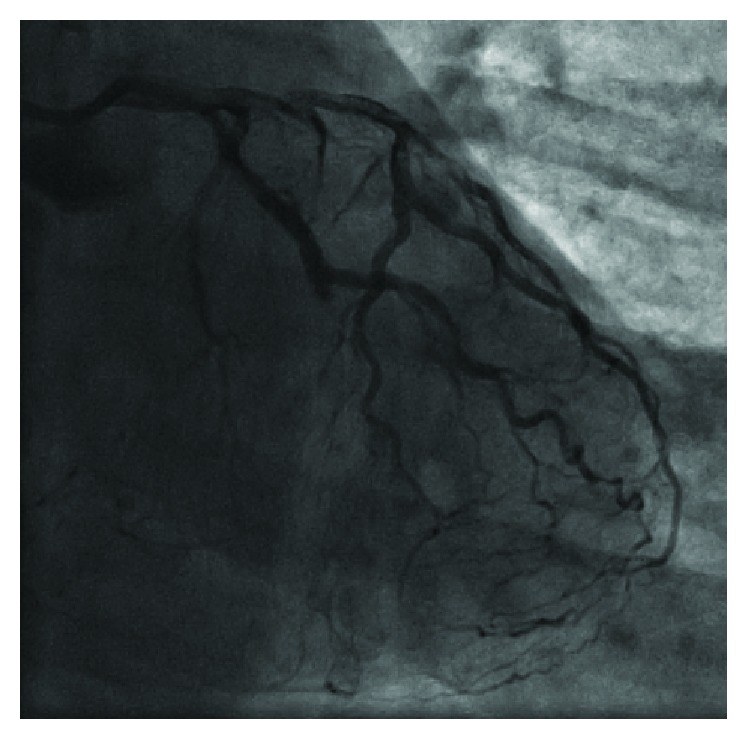
In-stent thrombosis of the left circumflex artery (LCx).

**Figure 6:**
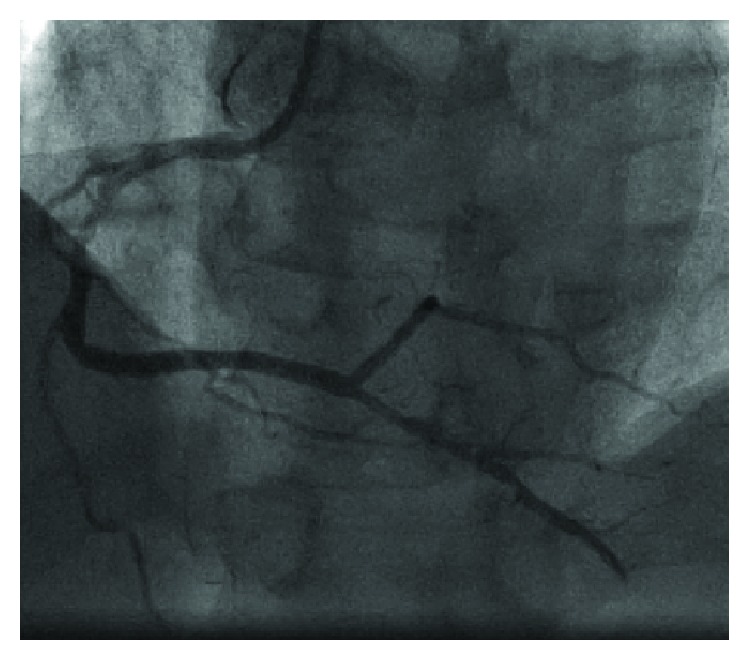
Recanalized right coronary artery (RCA) occlusion.

A 67-year-old woman with a history of asthma presented to the Emergency Department (ED) with chest pain lasting 3 hours before admission. The 12-lead ECG revealed myocardial infarction with ST segment elevation (STEMI) (elevation present in the inferior and V5/V6 leads) (Figures 1(a)–1(c)). The patient was hemodynamically stable with normal blood pressure and Killip status I. After applying a bolus dose of acetylsalicylic acid (300 mg) and ticagrelor (180 mg) orally, an urgent coronary angiography was performed which showed a middle segment left circumflex artery (LCx) occlusion and a collateralized total occlusion of the proximal segment of RCA. Three drug-eluting stents (DES) were implanted in the LCx, and due to unsatisfactory postprocedural TIMI flow (TIMI I), GP IIb/IIIa inhibitor eptifibatide was applied after the procedure (180 mcg/kg as a IV bolus—15,3 mg, followed by a continuous infusion of 2 mcg/kg/min up to 75 mg of eptifibatide in total) (Figures 2–4). Postprocedural ECG revealed satisfactory ST segment resolution, and the patient had no chest pain. Laboratory tests revealed elevation of cardioselective markers (admission values: hsTI 51 ng/L and creatine kinase 106 U/L; peak values during hospitalization, 18 hours after the intervention: hsTI 24100 ng/L and creatine kinase 1348 U/L). Echocardiography during the first day after procedure showed a preserved left ventricular ejection fraction (50%) with a inferoposterior wall hypokinesis, with no other significant pathology. The patient was treated with beta blocker, ACE inhibitor, and statin permanently as well as with a 100 IU/kg dose of low-molecule heparin (enoxaparin) twice a day during the first 4 days. On the 5th day of the hospitalization, the patient reported nonspecific chest discomfort, without cardioselective enzyme reelevation, but due to nonspecific changes in the inferior leads of the ECG, the new onset of the ischemia could not be excluded, so the coronary angiography was repeated. The second coronary angiography revealed CTO of the RCA and an in-stent thrombosis with occlusion of stents in LCx (Figure 5). Due to unsuccessful recanalization of the LCx using the guidewire and the TIMI I flow at the end of the first procedure, optimal anti-ischemic therapy was proposed including isosorbide-mononitrate, trimetazidine, ranolazine, in addition to ticagrelor, acetylsalicylic acid (ASA), nebivolol, ramipril, and atorvastatin. After the following four days of uneventful hospitalization, the patient was discharged with chronic therapy which included all the abovementioned medications. Three months later, at the planned outpatient follow-up visit, the patient presented with stable angina pectoris symptoms during moderate physical activity and a new coronary angiography was scheduled. After admission, coronary angiography was performed showing a spontaneous recanalization of the RCA, with a nonsignificant stenosis of the proximal-to-middle RCA segment, a 50% stenosis of the posterior descending artery, and no collaterals from left anterior descending artery (LAD) as well as persistent in-stent occlusion in the LCx with new collaterals from the first marginal artery (Figure 6). Dobutamine stress echocardiography was performed the day after the coronary angiography, showing no ischemia progression during testing in the RCA- and LCx-supplied myocardium. Due to stress echocardiography finding, no coronary intervention was indicated. Medical therapy was continued after dose optimization (80 mg of isosorbide-mononitrate daily instead of 40 mg and 1000 mg of ranolazine daily instead of 750 mg). Before discharge, an optimal ECG stress test was performed and no pain or ECG signs of ischemia were reported.

## 3. Discussion

We report a noninvasive CTO recanalization of the RCA. According to available data, a spontaneous recanalization of chronically occluded coronary arteries has been described anecdotally [5, 6]. All the cases (including ours) described CTO recanalization of the right coronary artery in the 60-year-old women. The majority of cases described CTO recanalization of carotid and peripheral arteries followed by discussion about the underlying mechanism [7–9]. In comparison to the reported cases with spontaneous CTO coronary artery recanalization, there are several specificities of our case. Firstly, no significant residual stenosis at the site of the previous CTO was noted, except a nonsignificant proximal-to-middle segment stenosis of the RCA [5, 6]. Secondly, the stimulation of the intrinsic fibrinolytic pathway by the anoxic tissue has been proposed as an underling mechanism of the discussed phenomenon [9]. In the reported case, acute therapy with low-molecule heparin and the GP IIb/IIIa inhibitor eptifibatide did not affect the chronic occlusion which was confirmed by a control angiography performed 5 days after the acute infarction. However, the addition of dual antiplatelet therapy in a chronic setting may be added a synergistic effect to intrinsic mechanisms and led to the recanalization in less than three months. It raises the question why the CTO was recanalized and the stents remained occluded. The fact that stents are “foreign” objects in the coronary arteries with a chance of acute and chronic thrombosis supports the hypothesis that thrombosis activated intrinsic fibrinolytic pathways, but stents in the LCx encouraged the thrombosis or made it difficult to intrinsic fibrinolysis to make the artery patent again. Thirdly, there is a suspicion that the RCA was a culprit lesion of the acute infarction. However, there are few arguments against the RCA occlusion as a culprit infarction lesion: the initial ECG showed a inferolateral ST segment elevation with a slightly higher elevation in the 2nd standard lead (II) than in the 3rd lead, without a significant “mirror” ST segment denivelation in the 1st standard lead and without elevation in the right precordial leads (RV4) (Figures 1(a)–1(c)); no ischemic pain and satisfactory ST segment resolution were present after the acute intervention on the LCx occlusion; there were already existing collaterals from LAD to RCA; and no medically induced reperfusion of the RCA occlusion was obtained (confirmed by a control angiography 5 days after the index event) after applying ticagrelor, acetylsalicylic acid, eptifibatide, and low-molecule heparin in the acute setting. It is possible that excessive antithrombotic and anticoagulant therapy gave a “bite” to the chronic occlusion of the RCA, leading to the intrinsic fibrinolytic mechanism and medical thrombotic suppression to melt the chronic occlusion. On the other hand, stopping the heparin and GPII/IIIa therapy in a still acute prothrombotic milieu led to thrombosis of the three implanted stents in the LCx. Additionally, it remains unclear why the patient reported angina between two hospitalizations, especially after performing a stress echocardiography where no ischemia progression was found. There might be an angiographic undetectable underlying microvascular disease which symptoms' regression can be related to the therapy changes (control ECG stress test revealed no ECG signs of ischemia nor clinical AP symptoms). Although intravascular ultrasound (IVUS) would have provided useful information, we were not able to perform it due to the reimbursement issues. Magnetic resonance imaging with gadolinium could not have been performed due to unavailability of the method in our hospital as well as the contraindication of adenosine administration due to the patient's history of asthma. In addition, another option could explain this sequence of events—prolonged coronary artery spasm which was repeated during the first two coronary angiographies. There are several facts pointing to the unlikelihood of spasm: presence of occlusion on two consecutive angiographies with 5-day difference while the patient was on full anti-ischemic therapy including nitrates and presence of collaterals to RCA supplying territory, no evidence of dynamic component of stenosis in angiograms during two different angiographies, as well as no evidence of spasm tendency on the third angiography. However, acetylcholine or ergonovine test during the last angiography, when RCA was found patent, which could have revealed the possibility of coronary artery spasm, was unfortunately not done [10, 11]. Lamm et al. [6] suggested that CTO is not a definitive state and collateral formation is just a bypass to the natural occlusion resolution. Perhaps it happens more often than we know because CTO patients are often treated with medications and usually no control angiographies are performed routinely. We hope that our data will help other authors to understand better the patho(physio)logy of the described unusual finding and encourage new basic studies in this area.
